# Concerns about the inverse relationship between baseline HBV DNA and on-treatment hepatocellular carcinoma risk. Reply.

**DOI:** 10.1172/JCI161425

**Published:** 2022-08-01

**Authors:** Won-Mook Choi, Young-Suk Lim

**Affiliations:** Department of Gastroenterology, Liver Center, Asan Medical Center, University of Ulsan College of Medicine, Seoul, South Korea.

**Keywords:** Hepatology, Hepatitis

## The authors reply:

We appreciate the letter from Liu et al. regarding our study (1).

First of all, the 2457 patients consecutively evaluated for eligibility in our study do not represent the total number of patients with chronic hepatitis B (CHB) during the ten-year enrollment period. Rather, those numbers were the remnants after excluding patients with cirrhosis, HBeAg negativity, previous history of malignancy or immunosuppressive agents, and follow-up duration of less than one year. We completely agree with and welcome validation of our results using independent cohorts with a larger sample size.

Second, we agree with the importance of other confounders, such as obesity, diabetes, drinking, and treatment response, in evaluating the risk of hepatocellular carcinoma (HCC) in patients with CHB. The result was consistent when the diabetes was further adjusted in 72.7% of the patients to whom the presence or absence of diabetes could be traced. Drinking is mostly based on self-reported data, which are prone to recall bias and cannot be fully guaranteed for data completeness and accuracy. Thus, we did not include drinking in the adjustment because of these possible biases. Obesity could not be adjusted any further because there were a lot of missing data in the study. However, a confounding factor that would affect the outcome must be unequally distributed among the groups being compared (2). In this case, there was no evidence from previous studies, and it is unlikely that the proportion of patients with obesity would be unequally distributed across the groups stratified by baseline HBV DNA levels. We did not include biochemical or virological response, since the analysis was based on the intention-to-treat principle.

Finally, subsequent data from the REVEAL cohort also showed a lower risk of HCC in patients with HBV DNA levels of greater than 10^7^ copies/mL than those with HBV DNA levels between 10^6^ and 10^7^ copies/mL (3, 4), in line with our study (5). A decreasing but considerable viral load of more than 5 log_10_ IU/mL may indicate the clonal emergence of hepatocytes that produce less virus. In addition, moderate serum HBV DNA levels were a risk factor for hepatic inflammation despite normal alanine transaminase levels and the absence of significant fibrosis (6), further contributing to the clonal hepatocyte repopulation. Hepatocarcinogenesis following the clonal expansion of hepatocytes and integration of HBV DNA into the host genome even with no significant fibrosis could be underway, which cannot be fully reversed by belated antiviral treatment. Moreover, considering the potent long-term efficacy and safety of current antiviral treatments that have a high genetic barrier to resistance and also a decrease in cost with the wide availability of generic drugs, antiviral treatment in immune-tolerant patients is cost-effective compared with delaying the treatment following the current clinical practice guidelines (7). Thus, we believe that early initiation of antiviral treatment may maintain the lowest risk of HCC in HBeAg-positive, noncirrhotic, adult patients with CHB, contributing to a decrease in global HCC incidence and HCC-related mortality.

## Version 1 08/01/2022

Electronic publication
